# Supplementary material for: HIVprotI: an integrated web based platform for prediction and design of HIV proteins inhibitors
Source: J Cheminform. 2018 Mar 9;10:12. doi: 10.1186/s13321-018-0266-y (PMC5845081; doi:10.1186/s13321-018-0266-y)
Supplement: Supplementary file 2 — Additional file 2. Source code of HIVProtI web server. [file 13321_2018_266_MOESM2_ESM.zip › HIVprotI_Source-code/hivprotI/jsme/JSME_depict_edit_toggle.html]

# Toggle depict - edit

This example shows how to switch back and forth between depict and edit mode.

---

Draw a chemical structure.  
Please click on the area below to edit the structure.

Edit structure
Hide editor
